# Queer-religious symbol analysis (QRSA): A semiotic method for reframing Hindu iconography

**DOI:** 10.1016/j.mex.2025.103689

**Published:** 2025-10-29

**Authors:** Preethika Shree AC, Chithra GK

**Affiliations:** Division of English - School of Social Sciences and Languages, Vellore Institute of Technology, Chennai 600127, India

**Keywords:** Gender fluidity, Hindu iconography, Queer-religious symbol analysis (QRSA), Queer theory, Sacred imagery, Visual semiotics

## Abstract

As sacred traditions face renewed scrutiny, there's a pressing need for methods that read religious iconography as fluid, resistant, and open to new meanings. This article presents the **Queer-Religious Symbol Analysis (QRSA)** method, a visual-semiotic analytical framework for the reinterpretation of Hindu religious iconography. This method enables the identification of latent meanings in deity forms, ritual symbols, and mythic visual narratives that have traditionally been interpreted within cisnormativity and heteropatriarchal paradigms. QRSA consists of five systematic phases: **(1) Iconographic Inventory**, cataloguing visual elements and attributes of religious images; **(2) Textual Contextualization**, linking iconography with scriptural and ritual sources; **(3) Queer Semiotic Reframing**, reinterpreting symbols through queer theoretical codes; **(4) Integrative Visual Analysis**, synthesizing symbolic, textual, and cultural layers; and **(5) Reflexive Documentation**, recording the analytical process with cultural accountability.

• Introduces a novel method for analyzing sacred Hindu symbols through queer theoretical lenses
• Enables inclusive reinterpretation of religious imagery without violating cultural sanctity
• Supports reproducible academic and curatorial practices for visual culture and heritage conservation.

## Graphical abstract

**Figure:**
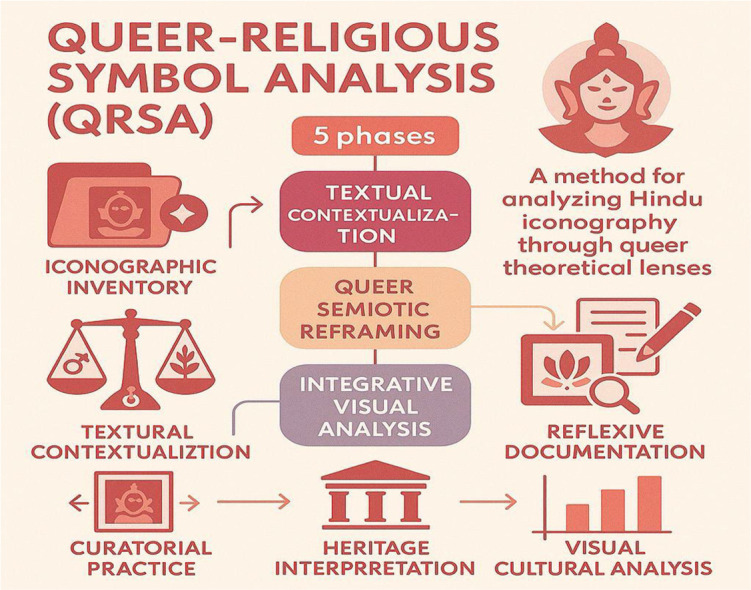

## Specifications table

**Table:** 

Subject area	Social Sciences
More specific subject area	Queer Religious Studies
Name of your method	Queer-Religious Symbol Analysis (QRSA)
Name and reference of original method	None. This is an original
Resource availability	Relevant resources include public domain images of Hindu deities, open-access translations of religious texts (e.g., Bhagavata Purana), and access to visual analysis tools (e.g., semiotic frameworks by Roland Barthes, Judith Butler’s gender performativity theory).

## Background

Religious iconography, especially within Hinduism, has long been a rich site for symbolic expression and layered meanings. Yet traditional interpretative frameworks often reflect heteronormative, patriarchal, and colonial biases that restrict the interpretive plurality of such imagery. This narrowing of meaning occludes the transformative, ambiguous, and subversive aspects inherent in many depictions of Hindu deitiesBose & Bhattacharyya [1]. Scholars like Doniger [2] and Kinsley [3], Kinsley [4] andS. Hawley [5] have revealed how Hindu mythological narratives are abundant in metaphors of sexual fluidity, divine androgyny, and gender nonconformity. However, these insights remain largely peripheral in both academic and public discourse due to sociocultural gatekeeping and institutional orthodoxy. Banerjee [6]. The gender-bending transformations of Vishnu into Mohini, the dual-gender form of Ardhanarishvara, and the fierce, untamed power of Kali and Bhairavi illustrate how Hindu mythology resists binary fixities and accommodates queer readings. These divine embodiments suggest that Hindu sacred art is not only compatible with, but at times constitutive of, non-binary spirituality. Yet, dominant narratives shaped by colonial-era scholarship (e.g., Monier-Williams) and postcolonial nationalist ideologies often sanitize or suppress such interpretations to uphold rigid moral and social norms [[7], [8], [9]]. The need to deconstruct these prevailing paradigms is urgent in a global climate increasingly aware of intersectionality and inclusivity [10]. Queer theory, pioneered by Butler [11], Eve Sedgwick (1993), and José Esteban Muñoz (2009), provides a valuable toolkit for interrogating fixed identities and exploring gender and sexuality as performative, relational, and historically contingent. Visual semiotics, particularly Barthes’ [12] analysis of signs and connotations, helps decode layers of religious imagery beyond conventional theological interpretation. The method emphasizes reflexivity and cultural sensitivity, avoiding the imposition of Western categories onto non-Western religious symbols while still advocating for non-normative interpretations. It aligns with decolonial and subaltern methodologies [13,14] that seek to displace dominant knowledge hierarchies and reclaim interpretive agency for historically marginalized voices. Despite these insights, current scholarship lacks a systematic and reproducible method to queerly analyze Hindu religious imagery. Prior work has highlighted instances of gender fluidity and divine androgyny, but these studies remain fragmented and interpretive rather than methodological. The present article addresses this gap by introducing Queer-Religious Symbol Analysis (QRSA), a structured semiotic method for decoding Hindu iconography. The aim of this research is to develop QRSA as a reproducible framework that bridges queer theory, visual semiotics, and Hindu sacred imagery. Ultimately, the QRSA method addresses an urgent methodological lacuna in visual religious studies: the lack of tools to read sacred iconography outside dominant cis-heteronormative paradigms. In doing so, it not only broadens academic inquiry but also contributes to the development of inclusive, dialogical approaches to faith, identity, and heritage in a rapidly pluralizing world. While QRSA is developed within the context of Hindu iconography, its broader significance lies in situating religious symbols as contested semiotic fields across traditions. Different religions engage LGBTQ+ identities, rituals, and symbols in divergent ways, yet share a common reliance on visual and material culture as sites of resistance and reimagination. Post-structuralist approaches make this comparative potential visible: for instance, Kassir [15] shows how LGBTQ+ Muslim activists mobilize the keffiyeh as a visual sign of queer pride and recognition in Lebanese performing arts. Such examples highlight how symbols across traditions are queered and re-signified, situating QRSA within a wider religio-gendered discourse. Similar insights emerge from Jewish-Queer material practices. Ben-Lulu [16], for instance, demonstrates how the mezuzah is reimagined as a “Pride Mezuzah” within American Jewish transgender communities, transforming a traditional religious object into a marker of queer belonging. Such examples illustrate how religious symbols across traditions—whether Hindu, Muslim, or Jewish—are mobilized to negotiate gendered identities through material culture.

## Method details

The Queer-Religious Symbol Analysis (QRSA) method is structured into five interlinked phases, each designed to critically reinterpret Hindu religious imagery through queer and semiotic frameworks. Table 1

**Table 1:** representation of the five interconnected phases of QRSA.

Phase Number	Phase Title	Focus Area
1	Iconographic Inventory	Visual documentation and classification of deity forms and symbols
2	Textual Contextualization	Scriptural and cultural positioning of the selected imagery
3	Queer Semiotic Reframing	Theoretical decoding using queer theory and semiotics
4	Integrative Visual Analysis	Synthesis of textual, visual, and symbolic interpretations
5	Reflexive Documentation	Positionality, interpretation notes, and ethical narrative reporting

Phase 1 **Iconographic Inventory** This foundational phase involves the systematic collection, documentation, and classification of religious imagery from Hindu visual culture. The goal is to create a comprehensive visual database that will serve as the basis for subsequent textual and semiotic analysis, particularly in identifying elements that defy rigid binaries of gender and sexuality [17].

### Objectives

**Fig. 1:**
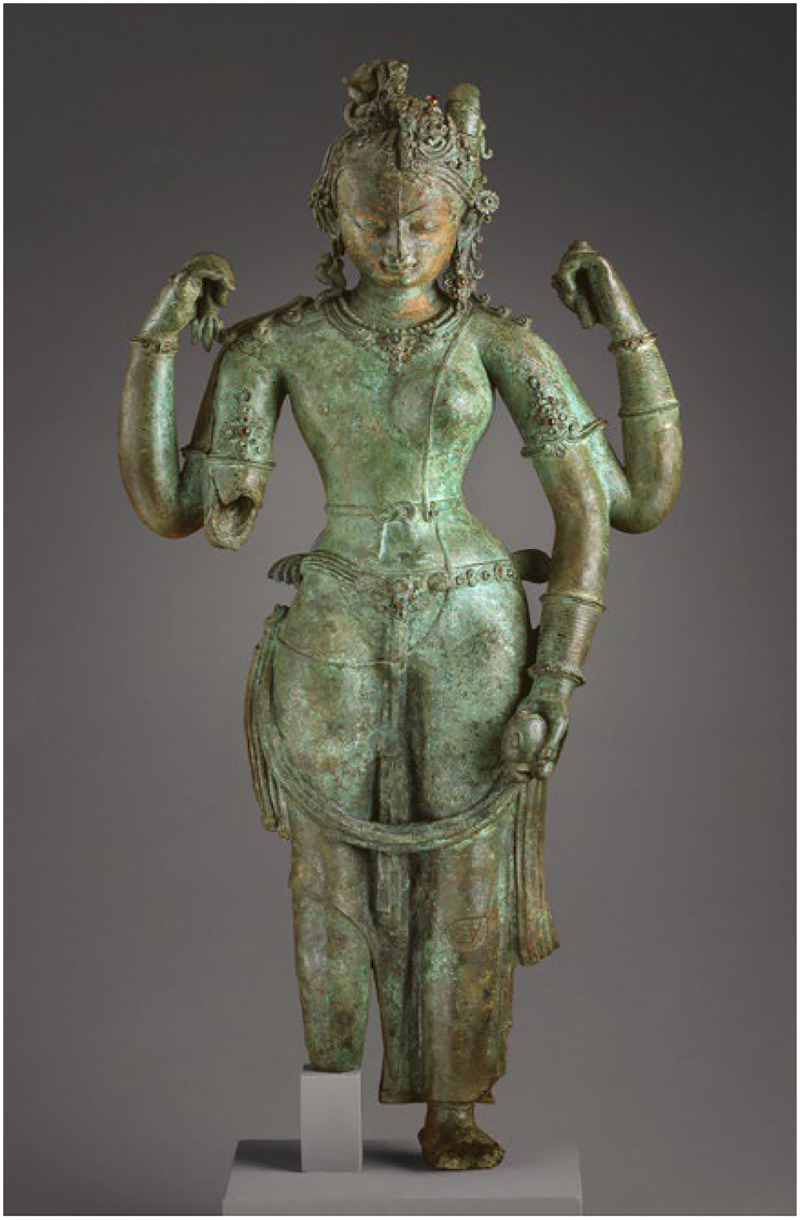
The androgynous form of Shiva and Parvati (Ardhanarishvara).

Source: Los Angeles County Museum of Art (LACMA)

• To gather diverse visual depictions of Hindu deities across regions, artistic styles, and historical periods.
• To document visual elements that may carry gendered, sexual, or symbolic connotations.
• To detect variations and divergences in iconographic patterns that could signal queer or fluid meanings. Fig. 1

### Materials and sources

• Public domain image repositories (e.g., Wikimedia Commons, museum archives)
• Temple sculptures, miniature paintings, manuscript illustrations, and devotional calendar art
• Field photographs and documentation from culturally significant rituals and festivals (with ethical considerations)

A 10th-century Nepalese bronze sculpture depicting Ardhanarishvara, the composite form of Shiva and Parvati. This image embodies the fusion of masculine and feminine energies, challenging binary gender constructs.

**Fig. 2:**
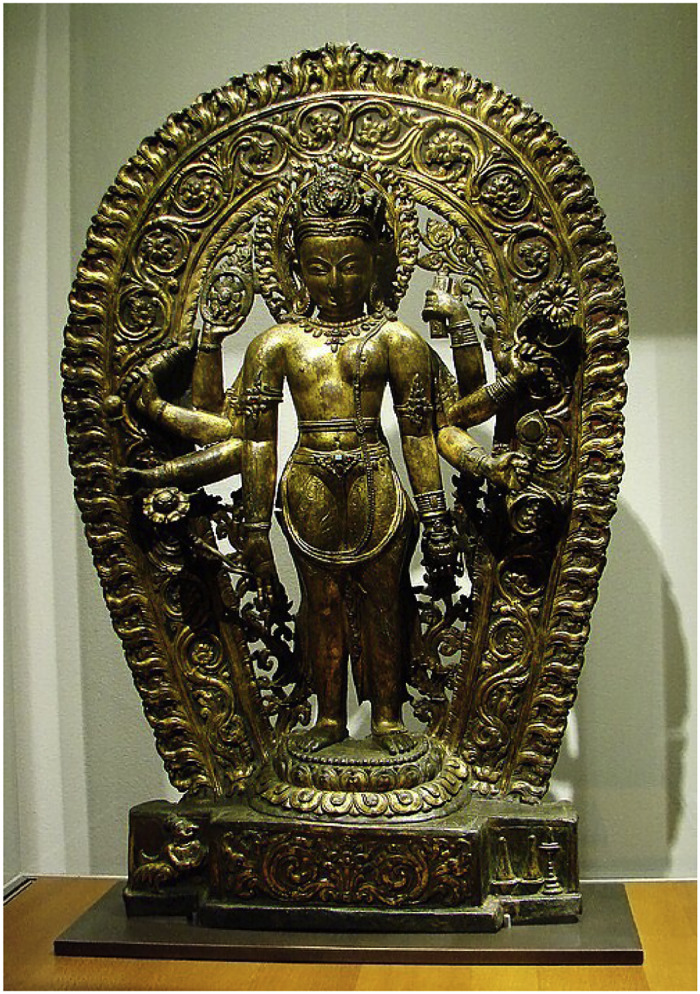
Vaikuntha-Kamalaja (or Lakshmi-Narayana).

Significance: Icon of non-binary embodiment and spiritual androgyny. Fig. 2

Source:https://en.wikipedia.org/wiki/Vaikuntha_Kamalaja

Like Ardhanarishvara, Vaikuntha-Kamalaja is depicted as half male and half female, split down the middle. The right half is the male Vishnu, illustrating his traditional attributes. The icon symbolizes the oneness or non-duality of male and female principles of the universe. Vaikuntha-Kamalaja is mentioned in few Tantric and iconographical texts.

### Steps

1. **Selection Criteria:** Begin by identifying images that exhibit iconographic ambiguity, gender hybridity, multiplicity in bodily representation, or atypical ornamentation. Particular attention is paid to depictions that blend male and female iconography, fluid sexual symbolism, or sacred eroticism.
2. **Cataloguing:** Develop a metadata-rich inventory using spreadsheets or qualitative data software. Each entry includes deity name, associated myth, region, period, source, medium, posture, mudras, attire, bodily features, accompanying figures, and any visual anomalies.
3. **Tagging for Queer Potential:** Use open and axial coding frameworks to tag visual traits with queer-theoretical significance. Tags may include categories such as "androgynous form," "cross-dressing deity," "same-gender intimacy," "non-dual posture," "liminal embodiment," or "intersex imagery."
4. **Preliminary Visual Grouping:** Images are grouped into typologies (e.g., composite deities like Ardhanarishvara, avatars like Mohini, or fierce goddesses like Kali). Cross-cultural and intra-traditional comparisons are made to trace shifts in representation across time and geography. Grouping helps identify recurring visual codes, marginal deviations, or regional aesthetics that disrupt normative readings.
5. **Archival Notation:** Each visual record is accompanied by its provenance, copyright status, publication context, and, if applicable, ritual or performative use. Notes on artist attribution, temple patronage, and sociopolitical background are added when available. This stage ensures that visual material is historically and ethically grounded.

**Fig. 3:**
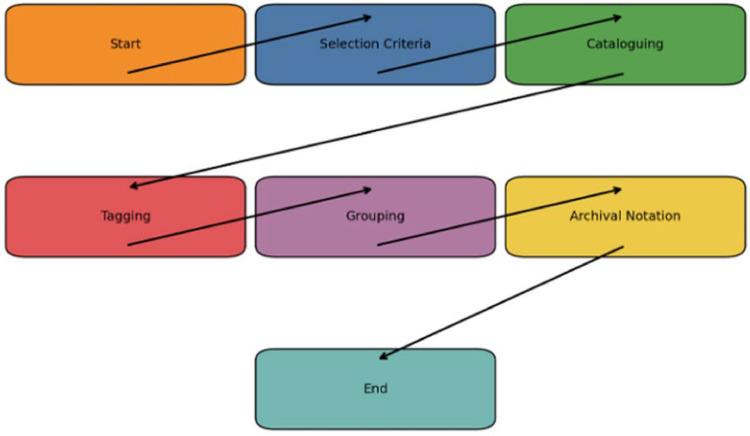
Flowchart of image documentation and tagging process.

As illustrated in Fig. 3, this phase is both data-driven and theoretically sensitive. It emphasizes the rigor of visual anthropology while incorporating the speculative and subversive insights of queer theory. By situating sacred images as both aesthetic artifacts and lived symbols, this phase sets the groundwork for decoding how the sacred intersects with queerness in visual culture.

Phase 2 **Textual Contextualization** This phase of QRSA focuses on situating the visual representations of deities within their corresponding textual and mythological frameworks. Rather than treating images as standalone artifacts, this stage anchors them in scriptural, oral, and regional narratives to uncover how meaning is mediated through both word and image [18].

**Fig. 4:**
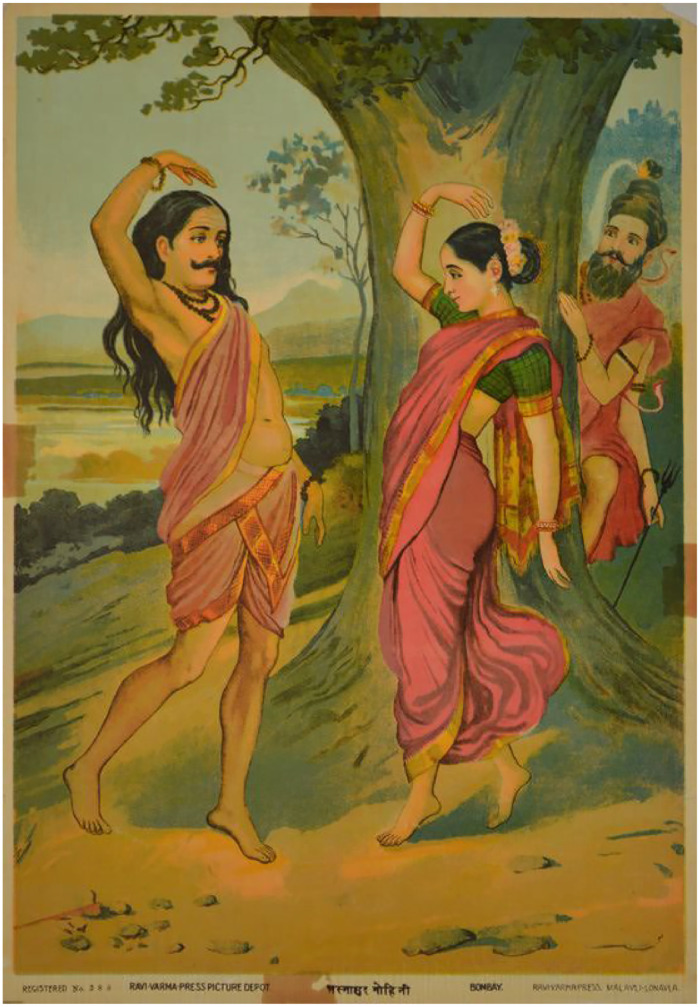
Raja Ravi Varma’s Mohini Bhasmasura.

The method involves correlating iconographic entries from Phase 1 with relevant sacred texts such as the Bhagavata Purana, Skanda Purana, Mahabharata, Ramayana, Tantric scriptures, and regional folklore. Textual sources are analyzed for interpretive flexibility, narrative layering, and depictions of gender/sexual transformation or ambiguity. The aim is not only to affirm the visual with textual legitimacy but to recognize how texts themselves encode or resist gender binaries. Fig. 4

Source:https://commons.wikimedia.org/wiki/File:Mohini_bhasmasura.jpg

This image visually represents the mythic episode of Vishnu’s transformation into Mohini to outwit Bhasmasura.

Significance: This image serves as a pivotal visual aid for: Text-Image Correlation, Gender Performativity, Ritual and Reception, Queer-Textual Tags. Table 2

**Table 2:** Visual depictions mapped to textual references.

Deity/Form	Textual Source	/Verse Description	Queer Interpretive Cue
Ardhanarishvara	Skanda Purana	Origin story of fused Shiva-Parvati form	Gender duality, sacred androgyny
Mohini (Vishnu)	Bhagavata Purana	Mohini's enchantment of demons and Shiva	Gender transformation, seductive fluidity
Bhairavi/Kali	Devi Mahatmya, Tantras	Slaying of demons, unbound hair, red tongue	Violent femininity, erotic rage
Krishna & Arjuna	Mahabharata (Udyoga Parva)	Krishna as charioteer and consort-like companion	Homoerotic devotion, emotional intimacy
Ila (Vishnu’s devotee)	Ramayana (later versions)	Transformation from male to female and back again	Non-linear gender identity
Aravan & Krishna	Tamil folk Mahabharata	Marriage of Krishna (as Mohini) to trans-fated Aravan	Ritualized queerness, divine drag performance

These pairings are not merely illustrative; they form the interpretive backbone of QRSA’s queer hermeneutics. For instance, the Ardhanarishvara form, visually composed with split male and female halves, invites reflection on non-binary embodiment. The Purana account frames this unity as divine harmony, which queer theory reads as a celebration of gender coexistence beyond dualistic hierarchies. Similarly, Mohini’s fluid form and strategic seduction operate not only as narrative devices but also as theological gestures toward embodied multiplicity. The Aravan and Krishna tale from Tamil traditions offers a striking example of how regional narratives preserve queer devotional frameworks often erased from mainstream discourse. The annual Koovagam festival in Tamil Nadu, in which transgender women ritually marry Aravan (symbolizing Krishna as Mohini), underscores the living textuality of queer myth [19].

Textual contextualization involves a layered process:

• **Canonical Anchoring:** Referencing classical scriptures to authenticate iconographic detail.
• **Comparative Commentary:** Drawing from bhashyas and regional versions to reveal narrative divergences.
• **Oral and Performative Interpretations:** Including folk tales, plays, dance-drama, and temple songs which carry encoded queer affective structures.

Ultimately, Phase 2 bridges visual semiotics and literary hermeneutics, anchoring imagery in living traditions while exposing the fluid potentials within sacred texts. It provides the linguistic, symbolic, and cultural scaffolding required for the semiotic reinterpretation carried out in Phase 3.

Phase 3 **Queer Semiotic Reframing** In this critical phase, QRSA applies queer theory and visual semiotics to reinterpret the symbolic language embedded in Hindu religious imagery. The goal is to uncover suppressed or overlooked meanings that challenge rigid gender binaries, heteronormativity, and fixed identity categories. Drawing from Roland Barthes’ model of denotation, connotation, and myth, this phase expands into a four-layered interpretive framework tailored to the complexity and polysemous nature of religious symbols. The process begins with identifying the **visual image (sign)**, such as Ardhanarishvara, Mohini, or Kali, as documented in Phase 1. At the first layer, the image is read at the level of **denotation**, focusing on the literal and descriptive elements: posture, attire, iconographic attributes, facial expression, and spatial arrangement. The second layer interprets **connotation**, incorporating culturally encoded meanings found in theological narratives, ritual performance, religious pedagogy, and inherited aesthetic conventions. These may include values like purity, balance, chastity, power, or divine roles assigned to masculine and feminine forms.

The third and most crucial layer is the **queer mythic reframing**. Here, the image is revisited through a queer theoretical lens to illuminate the latent meanings encoded through ambivalence, excess, and contradiction [20] drawing on Judith Butler’s theory of gender performativity, José Esteban Muñoz’s notion of disidentification, and Jack Halberstam’s work on failure and non-normativity [21], this interpretive act reveals how sacred symbols destabilize rather than affirm normative structures [22].

**Fig. 5:**
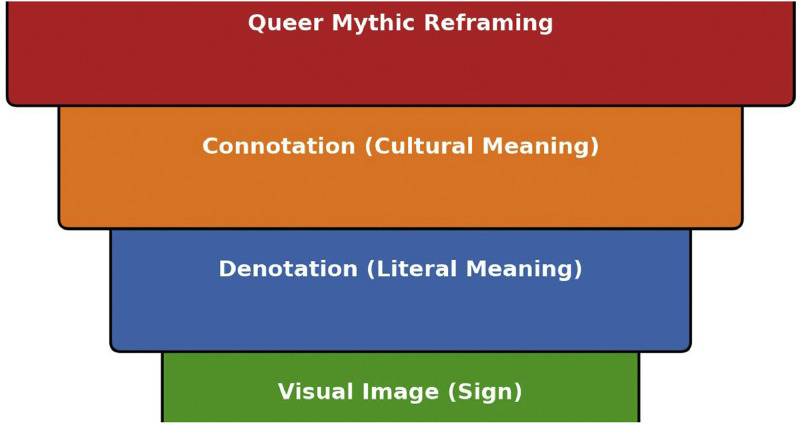
QRSA semiotic pyramid.

**Fig. 6:**
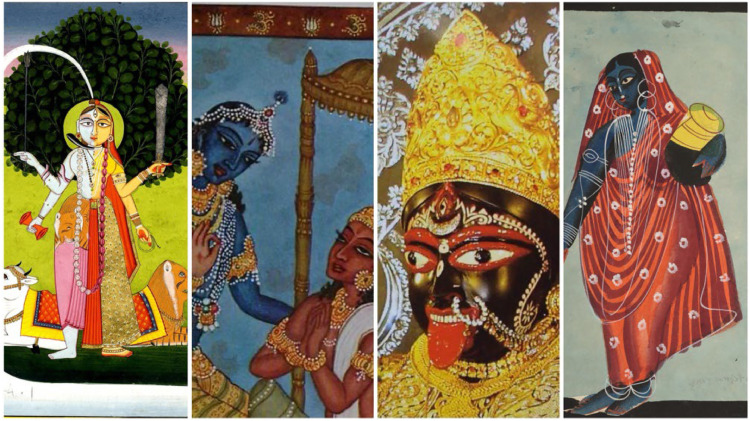
Iconic images for QRSA semiotic model.

This interpretive structure is visually represented in a conceptual pyramid:

Fig. 5. **QRSA Semiotic Pyramid: From Image to Queer Meaning** – This diagram visualizes the multi-layered analytical ascent in QRSA's semiotic method. The base denotes the physical icon or deity image. Above it lies the denotative layer (literal meaning), followed by the connotative layer (culturally encoded meanings), culminating at the apex with the queer mythic reframing. This interpretive summit challenges and transforms normative readings, asserting fluid and plural sacred meanings. This model enables scholars and curators to ethically reimagine sacred iconography through layered analysis. The ascent from literal to queer mythic reading mirrors both the interpretive journey and the expansion of symbolic possibility. The pyramid affirms that sacred images are not inert representations but semiotic fields of contestation and transformation. Fig. 6 and Table 3

**Table 3:** QRSA semiotic model to iconic images.

Visual Image	Denotation	Connotation	Queer Mythic Reframing
Ardhanarishvara	Half-Shiva, Half-Parvati figure	Union of masculine and feminine energies	Sacred non-binary embodiment; cosmic queerness
Krishna embracing Arjuna	Friendly gesture, intimacy	Devotional love, divine companionship	Homo-affective spirituality; divine queer desire
Kali's lolling tongue	Bloodlust or rage	Chaotic feminine energy, uncontrollability	Erotic refusal of docility; disruptive femininity
Mohini’s gaze	Alluring female figure	Seduction, maya (illusion)	Fluid gender performance; subversive femininity

This phase also introduces **queer coding**, the practice of identifying and interpreting specific visual signifiers such as gaze, adornment, color palette, spatial positioning, limb gestures (mudras), and bodily ambiguity that hint at fluid gender identity, queerness, or erotic liminality. In Kali’s iconography, for instance, the lolling tongue, blood-red palette, and transgressive stance signify not only destruction but also a refusal to comply with docile femininity. In Krishna’s affectionate positioning with Arjuna, the soft gaze and devotional proximity transcend devotional affection and open space for homo-affective interpretation.

**Affect theory** complements this process by allowing scholars to access the emotional vibrations and spiritual atmospheres within the image. Feelings of ecstasy, abjection, fear, desire, and transcendence become part of the queer analytical archive. These affects traditionally seen as apolitical or ephemeral are reframed as meaningful signs of spiritual intensity, often marking a departure from normative spiritual and gendered ideals.

Phase 4 **Integrative Visual Analysis** This phase synthesizes the insights generated from iconographic documentation (Phase 1), textual contextualization (Phase 2), and queer semiotic reframing (Phase 3) into a cohesive interpretive analysis. The objective is to construct a multidimensional understanding of Hindu religious iconography that is attentive to historical tradition while enabling transformative, inclusive readings through the QRSA lens. Integrative visual analysis is both comparative and dialogic. It positions each image within a matrix of meaning where visual form, textual myth, affective resonance, and theoretical engagement interact. This process enables scholars to trace how icons function across different interpretive registers, how form reflects narrative, how myth is reimagined through visual elements, and how both contribute to plural, queer readings of the sacred.

**Fig. 7:**
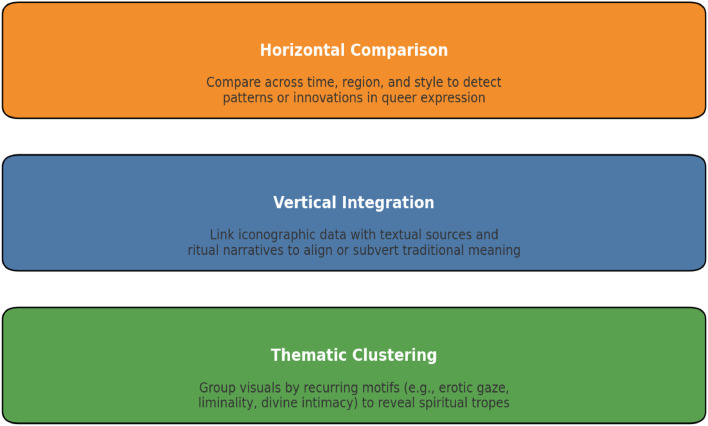
Integrative visual analysis in QRSA: three-tier comparative model.

To operationalize this phase, QRSA employs a **three-tier comparative model**, depicted below: Fig. 7

These layers allow for micro-level attention to detail and macro-level understanding of cultural patterning.

1. **Horizontal Comparison** involves studying variations of an image across regions, historical periods, and artistic forms. For example, comparing depictions of Mohini from Chola bronzes to 19th-century lithographs reveals a spectrum of gender presentation, from divine seductress to ambiguous liminal being.
2. **Vertical Integration** aligns visual data with mythological and ritual texts. For instance, Mohini's gaze in a mural might evoke themes from the Bhagavata Purana, but when viewed alongside tantric narratives, her seduction acquires an initiatory or transformative significance. This approach ensures that queer challenges rigid orthodoxy.
3. **Thematic Clustering** identifies recurring visual and emotional motifs across multiple images, such as merged bodies, divine embrace, hyper-femininity, or abjection. These motifs are then linked to broader queer spiritual tropes like ecstatic union, sacred eroticism, or embodied transformation.

This phase also insists on **analytical reflexivity**. Scholars are encouraged to record their interpretive choices, recognize their positionality, and remain attentive to ethical concerns surrounding appropriation, community engagement, and cultural translation. For example, interpreting Ardhanarishvara as a transgender symbol must be informed by indigenous gender categories and contemporary socio-religious dynamics, not imposed projections.

Phase 5 **Reflexive Documentation** The final phase of QRSA, Reflexive Documentation, emphasizes the importance of transparency, accountability, and scholarly ethics in the process of queer-religious interpretation. This phase ensures that each step of the analytical journey is recorded with precision, enabling both reproducibility and introspection.

Reflexive documentation involves systematically compiling analytical notes, interpretive rationale, cultural references, and visual annotations. It not only serves as a methodological archive but also foregrounds the subjectivity and positionality of the scholar within the interpretive process. This phase recognizes that the analysis of sacred images is shaped not only by texts and visuals but also by the interpreter’s theoretical lens, cultural location, and personal biases. Table 4

**Table 4:** Tripartite structure for documentation.

Documentation Element	Purpose	Format Examples
Interpretive Rationale	Records the logic behind each layer of interpretation	Analytical memos, coded notes, theoretical annotations
Cultural and Contextual Notes	Anchors interpretations in historical, ritual, and social contexts	Ethnographic observations, field notes, textual citations
Reflexive Statements	Articulates the scholar’s identity, assumptions, and ethical positioning	Narrative reflection, positionality statements

Reflexivity is not a disclaimer; it is an epistemological stance. Scholars are urged to critically examine how their interpretive acts might influence representations of queerness in religious contexts. For example, claiming divine queerness without grounding it in local cosmologies or communities may unintentionally exoticize or essentialize.

Ultimately, Reflexive Documentation reaffirms that QRSA is not only a framework for decoding religious symbols but a practice of relational ethics, cultural accountability, and scholarly humility. It invites ongoing dialogue rather than definitive claims, offering a living archive of how we see, know, and imagine the divine through queer lenses.

## Critical discussion

Previous scholarship on Hindu iconography has acknowledged elements of gender fluidity and divine androgyny [2,4,8]. However, these studies often emphasize isolated narratives or mythological motifs without providing a systematic framework for semiotic analysis. QRSA builds upon this body of research by offering a structured method that decodes symbols through queer theory and visual semiotics, enabling reproducibility and theoretical depth [23]. For example, Ardhanarishvara has long been interpreted as a theological representation of unity between masculine and feminine principles. QRSA extends this interpretation by reframing the figure as an icon of sacred non-binary embodiment, aligning with queer notions of fluid identity. Similarly, the queer reframing of Mohini and Aravan’s ritual marriage situates these figures within lived Hindu practices such as the Koovagam festival, where transgender communities ritualize divine drag and non-normative desire. These cases illustrate how QRSA not only engages with Hindu cultural contexts but also enriches them by exposing interpretive layers often obscured by cisnormative and patriarchal readings.

## Conclusion

By framing Hindu iconography within this wider comparative terrain, QRSA resonates with post-structuralist efforts to read religious symbols not as fixed dogmatic forms but as fluid semiotic practices. This opens possibilities for cross-religious dialogue, where sacred traditions can be reinterpreted through queer lenses that foreground shared struggles for recognition, fluidity, and inclusivity [24]. Further, by incorporating comparative cases such as the Pride Mezuzah in Jewish transgender communities [16], QRSA can be situated within a wider scholarly conversation on Queer Judaica, Islamic queer activism, and Hindu visuality [25]. These connections not only highlight the global resonance of queer-symbolic practices but also strengthen QRSA’s theoretical grounding in post-structuralist and material religion approaches [26]. In conclusion, the application of QRSA demonstrates that Hindu iconography is a fertile site for queer reinterpretation. Figures such as Ardhanarishvara, Mohini, and Kali reveal how sacred symbols destabilize binary constructs and open interpretive space for non-normative gender and sexuality. QRSA contributes by transforming fragmented queer readings into a systematic, reproducible method that can guide scholarship, pedagogy, and curatorial practice. In doing so, the method not only expands Hindu studies but also provides a model for inclusive approaches to religious visual culture more broadly.

## Limitations

The QRSA method, while innovative, has certain limitations. Its applicability is largely situated within South Asian Hindu iconography and may not seamlessly transfer to other cultural or religious contexts without adaptation. As a semiotic and affective framework, QRSA is inherently interpretive and shaped by the researcher’s positionality, which may introduce subjectivity. The method also depends on access to visual archives and reliable scriptural translations, which can be limited or uneven across regions and institutions [27]. Additionally, queer reinterpretations of sacred symbols may face resistance from traditional communities. Finally, despite safeguards, there remains a risk of over-queering visual material in the absence of supporting textual or ritual evidence. Although developed in a Hindu context, future studies might adapt QRSA to Jewish, Islamic, or other traditions where religious symbols are mobilized in queer communities.

## Ethics statements

This article does not contain any studies with human participants performed by any of the authors.

## CRediT authorship contribution statement

**Preethika Shree AC:** Writing – original draft, Methodology, Conceptualization. **Chithra GK:** Writing – review & editing, Supervision.

## Declaration of competing interest

The authors declare that they have no known competing financial interests or personal relationships that could have appeared to influence the work reported in this paper.

## Data Availability

No data was used for the research described in the article.

## References

[1] Bose B., Bhattacharyya S. (2007).

[2] Doniger W. (1999).

[3] Kinsley D. (1993).

[4] Kinsley D. (1988).

[5] Hawley J.S. (2001).

[6] Banerjee S. (2000).

[7] Guha-Thakurta T. (2004).

[8] Nanda S. (2009).

[9] Pattanaik D. (2003).

[10] Narrain A. (2004).

[11] Butler J. (1990).

[12] Barthes R. (1977).

[13] Spivak G.C., Nelson C., Grossberg L. (1988). Marxism and the Interpretation of Culture.

[14] Sunder Rajan R. (2005).

[15] Kassir, A.A.W. (2021). Divas of the night: Queer activism in Lebanese performing arts*.*

[16] Ben-Lulu E. (2025). Doorposts of inclusion: trans pride mezuzah as a marker of Jewish-queer space. Mater. Relig..

[17] Davis R.H. (1997).

[18] Dehejia V. (1997).

[19] Reddy G. (2005).

[20] Halberstam J. (2011).

[21] Muñoz J.E. (1999).

[22] Ahmed S. (2006).

[23] Kaushal A. (2022).

[24] Rao R. (2010).

[25] Tellis A. (2021).

[26] Dave N. (2012).

[27] Nair J. (2005).

